# Flubendazole inhibits glioma proliferation by G2/M cell cycle arrest and pro-apoptosis

**DOI:** 10.1038/s41420-017-0017-2

**Published:** 2018-02-14

**Authors:** Xumin Zhou, Jumei Liu, Jinming Zhang, Yong Wei, Hua Li

**Affiliations:** 1 Department of Pathogen Biology and Experimental teaching center of Preventive Medicine, Guangdong Provincial Key Laboratory of Tropical Disease, School of Public Health, Southern Medical University, Guangzhou, 510515 China; 2 Department of Respiration, Nanfang Hospital, Southern Medical University, Guangzhou, 510515 China

## Abstract

Flubendazole, FDA-approved anthelmintic, has been widely used in treating testinal parasites. In the recent years, Flubendazole has been reported to exert anticancer activities. On the other hand, little was known about the effects of Flubendazole on gliomas. Here we demonstrated a novel effect of flubendazole on glioma cells. We found that Flubendazole inhibited cell proliferation and promoted cell apoptosis of glioma cell lines in vitro, and suppressed tumor growth in xenograft models by intraperitoneal injection. However, Flubendazole might have no influence on cell migration. Mechanism study reaveled that Flubendazole caused cell cycle arrest in G2/M phase, which partly account for the suppressed proliferation. Consistently, Flubendazole induced P53 expression and reduced Cyclin B1 and p-cdc2 expression in glioma cells. In addition, Flubendazole promoted cell apoptosis by regulating the classical apoptosis protein BCL-2 expression. These observations suggest that Flubendazole exerts anti-proliferation and pro-apoptosis effects in Glioma through affecting the cell cycle and intrinsic apoptotic signaling, and indicate a novel utilization of Flubendazole in the treatment of Glioma.

## Introduction

Glioma accounts for 51.4% of all primary brain tumors, and is thus the most frequent primary malignant tumor of the adult central nervous system (CNS)^1, 2^. Glioma has high potential of proliferation and migration into healthy brain tissue^3^. The current treatment for glioma includes surgery, radiotherapy and chemotherapy, which have improved the survival rates, extremely frequent tumor recurrence is still inevitable^4^. Therefore, it is vital for the treatment of glioma to identify new carcinogenic pathways and therapeutic targets, and more efficient drugs are urgently needed because of the lack of valid chemotherapies, which could provid satisfactory clinical outcomes for glioma patients.

As a member of benzimidazole families, flubendazole contains the typical benzimidazole moiety. On the other hand, an added fluorine atom as the major structure makes it different from other benzimidazoles^5^. Flubendazole is a safe and efficacious anthelmintic drug, which is widely used for anthelmintic to human, rodents and ruminants^5–9^. Recent studies showed that flubendazole played a novel role in inhibiting cell growth in colon cancer, breast cancer, leukemia, and intestinal cancer^10–13^. What’s more, neuroblastoma was identified as highly flubendazole-sensitive cancer^14^. However, the specific roles of flubendazole in glioma remain unclear. According to the report^15^, we know that the toxic action of benzimidazole compounds would not be reduced by the blood–brain barrier. To clarify that issue, we investigated the effects of flubendazole on tumorigenesis and progression in glioma in this study.

## Results

### Flubendazole inhibits the cell proliferation in human glioma cells

**Fig. 1:**
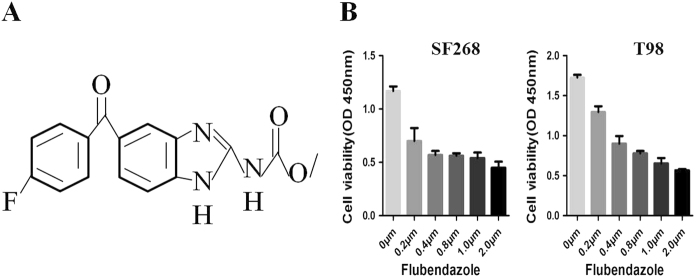
**a** Chemical structure of flubendazole. **b** The changing 50% inhibitory concentration of flubendazole fitting in SF-268 and T-98G cells

**Fig. 2:**
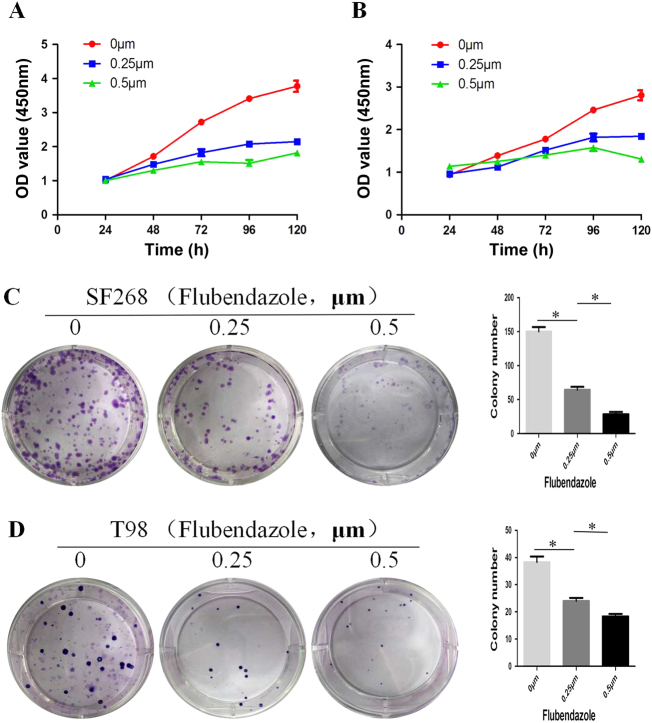
Flubendazole inhibits cell proliferation in human glioma cells. **a**,** b** Flubendazole inhibits the proliferation of SF-268 (**a**) and T-98G (**b**) cells as detected by CCK-8 assays. **c**,** d** Representative images of the SF-268 and T-98G cell colonies after treatment of 0, 0.25, and 0.5 μm flubendazole were shown. Each bar represents the mean ± SD of three independent experiments. *P < 0.05

The chemical structure of flubendazole was shown in Fig. 1a. To gain insight to the possible cytotoxic effect of flubendazole in human glioma cells growth, SF-268 and T-98G cells were exposed to the increasing concentration of flubendazole (from 0 to 2 μM) for 24 h, respectively. The changing 50% inhibitory concentration of flubendazole fitting in SF-268 and T-98G cells were about 0.4 and 0.5 μM (Fig. 1b). CCK-8 assay showed that flubendazole significantly reduced cell viability in glioma cells (Fig. 2a, b). At the same time, colony formation assay indicated that flubendazole inhibited the clonality of SF-268 (*P* < 0.001) and T-98G cells (*P* < 0.001) (Fig. 2c, d).

### Flubendazole affacts tumorigenesis of SF-268 cells in vivo

**Fig. 3:**
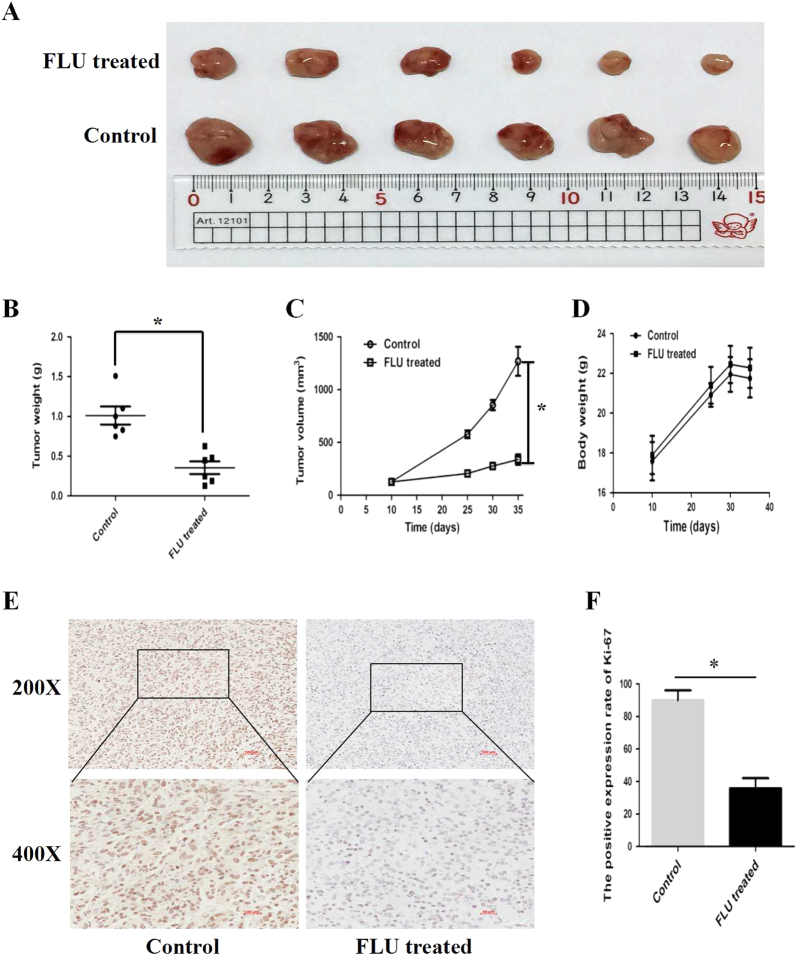
Flubendazole affacts tumorigenesis of SF-268 cells in vivo. **a** Image of tumors dissected from 12 nude mice (*n* = 6 for each group). SF-268 cells were incubated into the right armpit regions of each mouse. After injection 10 days, mice were randomly distributed into two groups, flubendazole-treated group (25 mg/kg per day) and control group. **b** The tumors weight were weighed and presented by scatter plot. **c** Tumor volumes were measured on the indicated days and drew into the tumor growth curves. **d** The body weight of each mouse was monitored during the experiment. **e** Representative immunohistochemistry staining images of Ki-67 from tumor tissues in each group. **f** Mean positive rates ± SD were shown (*n* = 6). The data represent the mean ± SD of three independent experiments.*P < 0.05 (vs. control)

As flubendazole expressed anti-proliferation activity on glioma cells in vitro, we further suspected whether flubendazole inhibited tumorigenicity in vivo. To indentify the effect of flubendazole on tumor growth, we performed tumorigenesis assays in the xenograft tumor model. 5 × 10^6^ SF-268 cells were inoculated into the right armpit regions of each mouse. When the tumors developed for 10 days (~120 mm3), mice were randomly distributed into two groups to receive flubendazole (25 mg/kg, once daily) and vehicle control intraperitoneally. After 25 days of treatment, we found that the subcutaneous tumors of flubendazole-treated group were smaller and lighter than that of control group (*P* < 0.005). However, the body weight showed no obvious difference between two groups (*P* > 0.05). Furthermore, the behavior, feeding pattern and overall activity of mice did not show significant changes. To further clarify the inhibitory effect of flubendazole on tumor growth, immunohistochemical analysis was used to detect the expression of Ki-67 in paraffin-embedded mice tumors. As depicted in Fig. 3e, the expression of Ki-67 in flubendazole-treated group was significantly lower than that in control group. All these results demonstrated that flubendazole significantly inhibited tumor growth in xenograft model with favorable toxicology profiles.

**Fig. 4:**
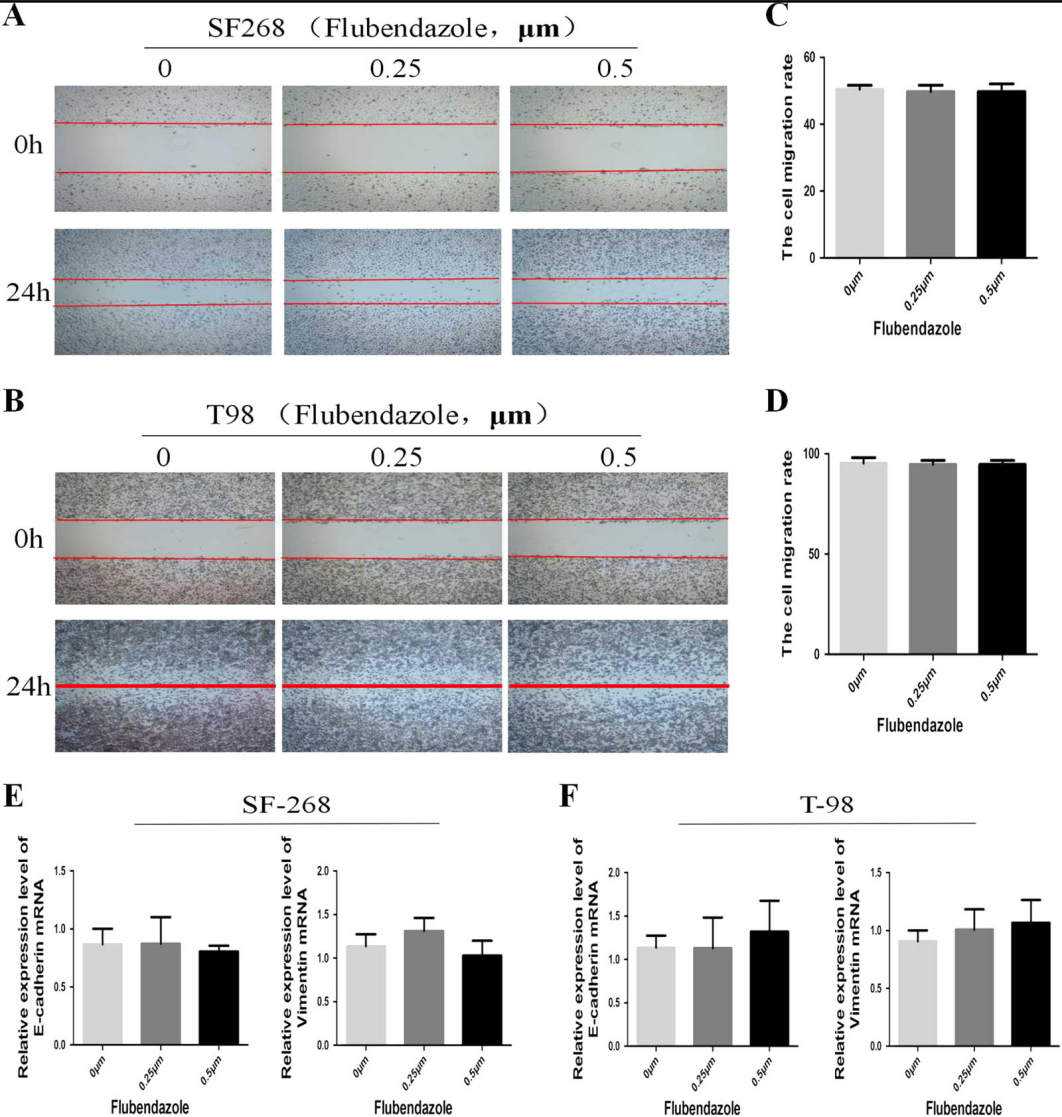
Flubendazole might have no influence on cell migration. **a**,** b** SF-268 and T-98G cells were treated with indicated concentration of flubendazole or vehicle control for 24 h, and the effects on cell migration were determined with cell scratch test.The cell migration of both cell lines. **c**,** d** Quantitative results. Values are the mean of triplicate samples from a representative experiment. *P < 0.05. **e**,** f** The RT-PCR analysis of the expression of epithelial marker (E-cadherin) and mesenchymal marker (Vimentin) after treatment of 0, 0.25, and 0.5 μm flubendazole for 24 h. *P* > 0.05

**Fig. 5:**
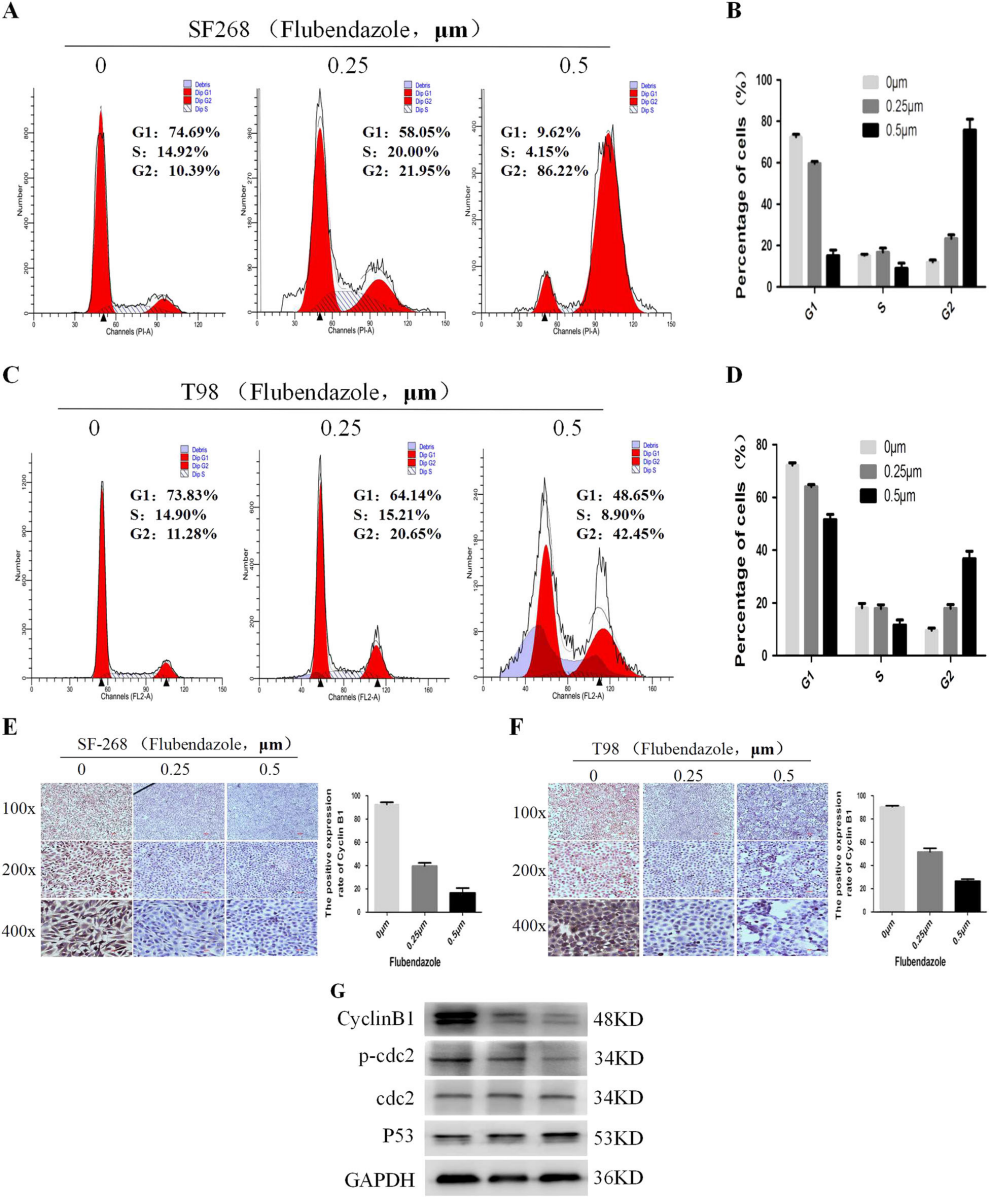
Flubendazole arrests cell cycle at G2/M phase. **a**,** b** Flow cytometric analysis of cell cycle arrest after treatment of 0, 0.25, and 0.5 μm flubendazole in SF-268 and T-98G cell lines. **c**,** d** Mean percentage of cells ± SD were shown (SF-268 and T-98G). *P < 0.05. **e**,** f** The expression levels of Cyclin B1 protein in IHC after treatment of 0, 0.25, and 0.5 μm flubendazole for 24 h (SF-268 and T-98G). The data represented as mean ± SD of three independent experiments. *P < 0.05. **g** Western blotting analysis of the expression of Cyclin B1, P53, and cdc2. GAPDH was used as a loading control

### Flubendazole might have no influence on cell migration

Then, we used the wound healing assay to valuate the effect of flubendazole on cell migration. The cell migration rate between the flubendazole-treated group (0.25 and 0.5 μm) and vehicle control group showed no obvious difference (*P* > 0.05) (Fig. 4a, b, c, d). In addition, we examed the expression of epithelial–mesenchymal transition (EMT), which is closely correlated with cell migration. Results revealed that the expression of mesenchymal markers (Vimentin) and E-cadherin did not show significant changes after the treatment with 0.25 and 0.5 μm flubendazole for 24 h compared to vehicle control (*P* > 0.05) (Fig. 4e, f). These data demonstrated that flubendazole might have no influence on cell migration of SF-268 and T-98G.

### Flubendazole arrests the cell cycle at G2/M phase

The results above showed that flubendazole inhibited the proliferation of SF-268 and T-98G cells. Regarding the impact on the cell cycle, which acts the main role in cell proliferation, we performed flow cytometry. After analyzing the cell cycle distribution of SF-268 and T-98G, we found that the percentage of G1 phase cells was decreased from 72.56 ± 2.14, 72.21 ± 1.69 to 59.68 ± 1.65, 64.13 ± 1.35 and 15.14 ± 4.81, 51.58 ± 3.41 with the increasing concentration of flubendazole (*P* < 0.001). On the contrary, the percentage of G2 phase cells was increased from 12.16 ± 1.53, 9.63 ± 1.53 to 23.46 ± 3.08, 17.98 ± 2.38 and 75.83 ± 9.01, 36.8 ± 4.90 (*P* < 0.001) (Fig. 5a, b, c, d). It has been widely known that the protein Cyclin B1 is closely correlated with G2 phase arrest, we examed its expression in SF-268 and T-98G cells after treating with the flubendazole by immunocytochemistry (Fig. 5e, f) and western blotting^16^. We found that flubendazole downregulated the expression of Cyclin B1 and p-cdc2, while upregulated the expression of P53. ALL of these data revealed that flubendazole induced cell cycle arrest in G2 phase (Fig. 5g).

### Flubendazole promotes the apoptosis of glioma cell lines

**Fig. 6:**
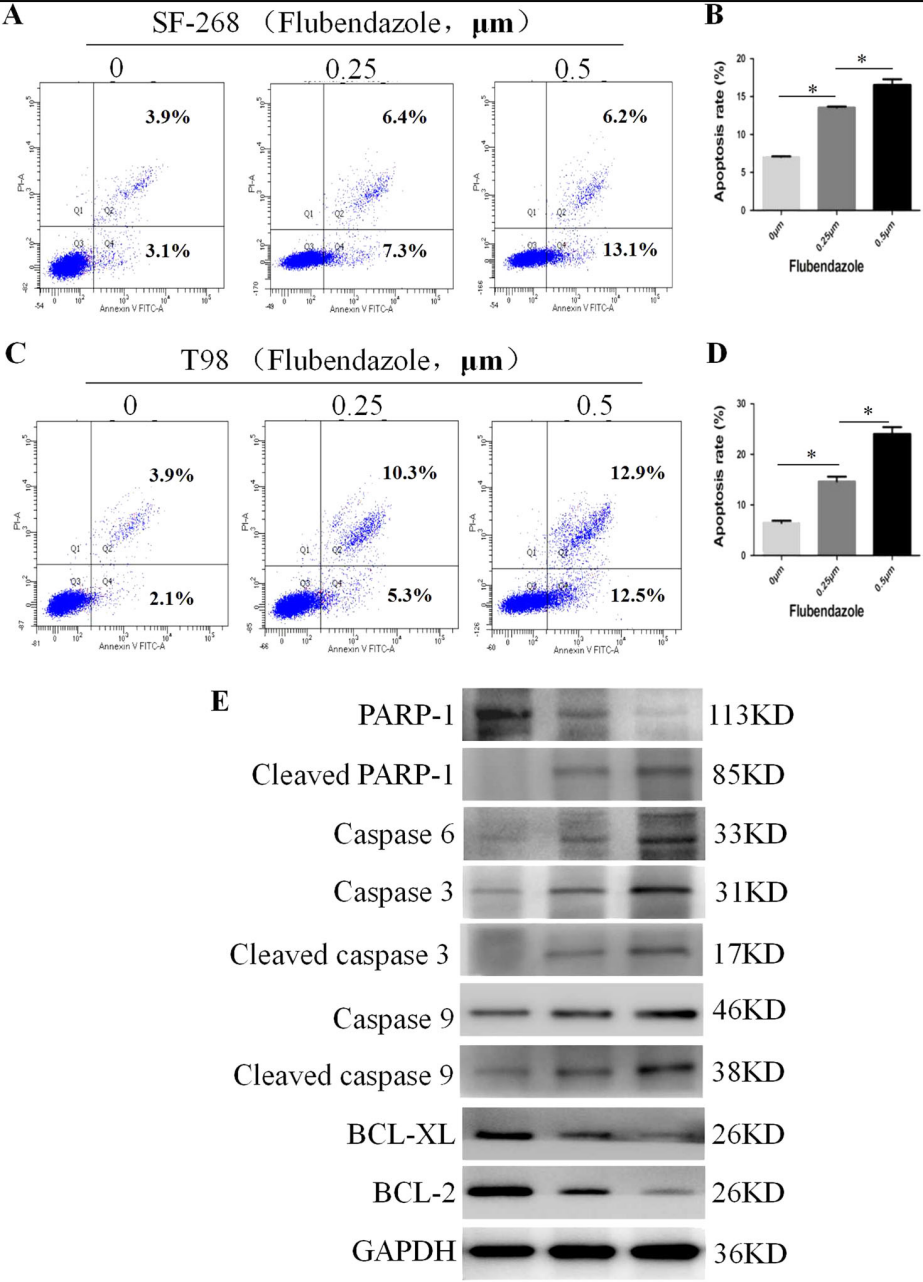
Flubendazole promotes the apoptosis of glioma cell lines. **a**,** b** Flubendazole promotes the apoptosis of SF-268 (**a**) and T-98G (**b**) cells, which were analyzed by flow cytometric using Annexin V-FITC/PI staining. **c**,** d** Statistical results were represented as mean ± SD of three independent experiments. *P < 0.05. **e** The expression of Bcl-2, caspase-3, and caspase-9 was identified by western blotting after treatment of 0, 0.25, and 0.5 μm flubendazole for 24 h (SF-268 and T-98G). GAPDH was used as a loading control

We then examined the involvement of flubendazole in the apoptosis of glioma cells through treating with the increasing concentration of flubendazole (from 0 to 0.5 μm). As shown by flow cytometry analysis, the apoptotic rates in SF-268 and T-98G cells of flubendazole-treated group were significantly higher than those of vehicle control group (*P* < 0.005). To our knowledge, the intrinsic apoptotic pathway acts an important role in cell apoptosis. Thus, we measured the expression of Bcl-2/caspases pathway after treating with flubendazole. Results showed that flubendazole downregulated the expression of BCL-2 and BCL-xl, whereas upregulated the expression of Cleaved PARP-1, Cleaved caspase-3 and Cleaved caspase-9 (Fig. 6e). All these results indicated that flubendazole induced the SF-268 and T-98G cells apoptosis through the intrinsic apoptotic pathway.

## Discussion

Flubendazole has been extensively studied for the treatment of parasites in animals and humans with favorable toxicology profiles^17–23^. For example, the LD50 is >5000 mg/kg flubendazole after oral administration and >400 mg/kg flubendazole after intraperitoneal administration in rats, mice, and guinea pigs^13^. In addition, no toxicity from the drug was noted in humans that received doses of 40–50 mg/kg per day flubendazole for 10 days. Likewise, there is no adverse effect that the alveolar echinococcosis patients received up to 50 mg/kg flubendazole daily for up to 24 months^24^. Consistently, our results displayed that the gaining weight of nude mice show no obvious difference between the flubendazole-treated group and the control group, indicating less toxicity to the mice.

In the present study, we demonstrated that flubendazole, a FDA-approved anthelmintic drug, inhibited glioma cells proliferation in vitro and in vivo. Tumor progression of reflects dysregulation of cell apoptosis or the cell cycle, which results in the limitless proliferation in cancer^25, 26^. The major regulatory checkpoint in this process is the transition of cell cycle from G1/S phase to G2/M phase^27^. In our research, flow cytometric analysis revealed that flubendazole arrested cell cycle at G2/M phase. During the cell cycle, cyclin B1 acts a positive regulatory role in G2/M phase, which triggered the mitosis. The repression of both cyclin B1 and cdc2 would lead to overriding p53-mediated G2/M arrest^16, 28^. Thus, we evaluated the expression of proteins which are involved in regulating cell cycle transition from G2 phase to M phase after treatment of flubendazole. Our results showed that flubendazole inhibited the expression of cyclin B1 and cdc2 while enhancing expression of p53. These data partly shed light on the way in which flubendazole inhibits proliferation of glioma cells.

It is reported that flubendazole affacted the caspase family, which are absolutely essential for the initiation and execution of cell apoptosis^10, 29–31^. Also, the apoptosis in glioma cells is linked to the activation of Caspase-3, which plays an important role in the intrinsic signaling pathway^32, 33^. Futhermore, Bcl-2 serve as an important regulator downstream of caspase signaling^34^. Therefore, we examed the expression of those proteins by western blotting. In our study, the results showed that flubendazole increased apoptosis among glioma cells via downregulating the expression of Bcl-2, Bcl-xl, and PARP-1, while upregulating the expression of caspase-3, caspase 6 and caspase-9. These data suggest that flubendazole isinvolved in regulating the Bcl-2/caspase/PARP-1 signaling pathway.

To sum up, results from our study demonstrate that flubendazole appears to exert anti-proliferation and pro-apoptosis effect in glioma cells. These actions of flubendazole in glioma cells are most likely mediated via the cyclin B1/cdc2/p53 and Bcl-2/caspase/PARP-1 signaling pathways. These data provided a strong evidence for the potential utilization of flubendazole in the clinical treatment of glioma.

## Materials and methods

### Reagents

Flubendazole was purchased from. Flubendazole was prepared in dimethyl sulfoxide for a final concentration of 1 mmol/L. In all experiments, the final DMSO concentration was <0.1% and DMSO alone had no demonstrable effect on cultured cells.

### Cell culture

Human glioma cell lines SF-268 and T-98G were derived from American Type Culture Collection (ATCC). Cells were maintained in 1640 medium (Gibico, China) supplemented with 10% fetal bovine serum (Gibico, China) and incubated under 5% CO_2_ at 37 °C.

### Cell viability analysis

SF-268 and T-98G were seeded into 96-well plates and cultred with various doses of flubendazole for 24 and 48 h, respectively. The cell growth and viability were measured using the Cell Counting Kit-8 (CCK-8) assay (TongRen, China) according to the manufacturer’s protocol and as previously described^35^. The optical densities (OD) were measured at absorbance 490 nm. Three duplicate wells should be plated for each cell group.

### Colony formation assays

To identify the influence of flubendazole on the ability of SF-268 and T-98G cells to form colonies, 1000 cells were seeded into 6-well plates and incubated with 0, 0.25, and 0.5 μm flubendazole for 9 days, respectively. After 9 days, cells were washed three times with cold PBS, and then fastened with 4% paraformaldehyde for 1 h. The colonies were stained with hematoxylin for 30 min, and then counted using a microscope.

### Cell cycle analysis

SF-268 and T-98G cells were treated with indicated concentration of flubendazole for 36 h, and then collected in PBS and fixed in 70% ice-cold ethanol for overnight at 4 °C. After washing in cold PBS, the cells were suspended in 200 μl PBS and incubated with 20 μl Rnase A for 30 min at 37 °C the next day. The mixture was then stained with 400 μl PI in shade for 1 h. The cell cycle phases were detected by flow cytometry system (BD FACSCalibur).

### Cell apoptosis analysis

To analyze whether flubendazole affects the apoptosis, SF-268 and T-98G cells exposed to indicated concentration of flubendazole for 36 h, and then collected and stained with annexin V-FITC and propidium iodide (PI) according to the manufacturer’s instructions. The apoptotic percentages of the treated cells were analyzed by flow cytometry using a BD FACSCalibur system.

### Tumor growth in xenografts

A total of 12 nude mice (4 weeks) were gained from the experimental animal center of southern medical university (GuangZhou, China) and fed under specific pathogen free conditions. 5 × 10^6^ SF-268 cells were inoculated into the right armpit regions of each mouse. After injection 10 days, mice were randomly distributed into two groups to receive 25 mg/kg flubendazole in 0.9% NaCl and 0.01% Tween-80 or vehicle control (0.9% NaCl and 0.01% Tween-80) once daily by intraperitoneal injection. After administration of flubendazole or vehicle for 25 days, all mice were killed, and tumor weight and tumor volume were immediately measured, respectively.

### Cytochemistry

Cells were cultured on slide glasses for one night and fixed with 4% paraformaldehyde for 1 h. After washed three times with PBS, cells were incubated with 3% H_2_O_2_ for 30 min and blocked with 5% BSA for 1 h at 37 °C. Then, cells on slide glasses were incubated with cyclin B1 primary antibodies overnight at 4 °C and incubated with appropriate secondary antibodies for 1 h at 37 °C. Finally, signal was detected with DAB and nuclei were counterstained with hematoxylin.

### Immunohistochemistry and evaluation criteria

For immunohistochemistry (IHC), tumor tissues were fixed with 10% formalin, dehydrated and embedded in paraffin. Then 2.5-μm sections were prepared with a microtome. The sections were deparaffinized, rehydrated and the endogenous antigen was retrieved by autoclave for 10 min in citric-acid buffer (10 mM citrate buffer, PH6.6). The slides were then incubated with anti-Ki-67 primary antibody at 4 °C overnight and then HRP-conjugated secondary antibody at 37 °C for 30 min. Signal was detected with DAB and nuclei were counterstained with hematoxylin. Ki-67 expression in cancer cells of the tumor tissues was evaluated by the total scores of intensity and the percentage of stained cells. Intensity of stained cells was scored as follows: (1) 0—lack of staining (−), (2) 1—weak staining (+), (3) 2—moderate staining (++) (4) and 3—strong staining (+++). The percentage of stained cells was recorded as 1–25% or less, 2–26 to 50%, 3–51 to 75%, (4) and 4—greater than 75%. Total scores of stained cells ranged from 0 to 12. All sections were defined as having low expression—0 to 7 or high expression—8 or greater by semiquantitative score. Slides were assessed by 2 pathologists.

### Real-time RT-PCR and western blotting analysis

Total RNA was extracted using TRizol reagent (TAKARA, China), and first-strand cDNA was synthesized with the PrimeScript RTReagent Kit (TAKARA, China) according to the manufacturer’s recommended instructions. Reverse Transcription was carried out with the SuperScript First-Strand Synthesis System for RT-PCR (Invitrogen, Carlsbad, CA) according to the manufacturer’s protocol. Real-time RT-PCR was carried out using complementary DNA and SYBR-Green II (TAKARA, China). The data were normalized to the housekeeping gene GAPDH and counted using the 2−ΔΔCT method. RT-PCR was performed on the ABI PRISM 7500 Sequence Detection System (Applied Biosystems) and repeated at least three times. Primers were designed using Primer Express software, and their sequences were: E-cadherin, sense, 5′-TTGTGGCCCTGTCCAGAGAATT-3′; anti-sense, 5′-GTTTGTGTAGTCCCAAGTATCCTG-3′; Vimentin, sense, 5′-TTGTGGCCCTGTCCAGAGAATT-3′; anti-sense, 5′-GTTTGTGTAGTCCCAAGTATCCTG-3′; GAPDH, sense, 5′-AGAAGGCTGGGGCTCATTTG-3′; anti-sense, 5′-AGGGGCCATCCACAGTCTTC-3′.

Cells were washed three times by cold PBS and lysed by lysis buffer (KaiJi, China) containing protease inhibitor cocktail. The concentration of protein was determined by bicinchoninic acid method. For western blotting analysis, proteins were separated by 10% SDS-polyacrylamide gel and was then transferred onto 0.22 μm pore-size PVDF membrane. After blocking in 5% skimmed milk in PBST, the membrane was incubated with appropriate primary antibodies at 4 °C overnight. Then, the membrane was washed 5 min for six times, followed by incubation with HRP-linked secondary antibody for 2 h at room temperature. GAPDH was used as an internal control. Protein blots were detected by exposing chemiluminescent HRP substrate to film.

### Statistical analysis

All of the experiments were repeated at least three times and the data were showed as means ± S.D. The student’s t-test were used to compare the differences between two independent samples. IC50 was calculated by a nonlinear regression using one site competition curve. All statistical analyses were carried out with SPSS 20.0. *P* < 0.05 was considered statistically significant.

## References

[1] Goodenberger ML, Jenkins RB (2012). Genetics of adult glioma. Cancer Genet..

[2] Jansen M, Yip S, Louis DN (2010). Molecular pathology in adult gliomas: diagnostic, prognostic, and predictive markers. Lancet Neurol..

[3] Armstrong TS, Wen PY, Gilbert MR, Schiff D (2012). Management of treatment-associated toxicites of anti-angiogenic therapy in patients with brain tumors(). Neuro. Oncol..

[4] Zhang J-q YaoQh, Kuang Y-q MaY, Yang L-b, Huang Hd (2014). Prognostic value of coexistence of abnormal expression of micro-RNA-200b and cyclic adenosine monophosphate-responsive element-binding protein 1 in human astrocytoma. Hum. Pathol..

[5] Ceballos L (2011). Comparative performances of flubendazole and albendazole in cystic echinococcosis: ex vivo activity, plasma/cyst disposition, and efficacy in infected mice. Antimicrob. Agents Ch..

[6] Ceballos L (2013). A pharmacology-based comparison of the activity of albendazole and flubendazole against Echinococcus granulosus metacestode in sheep. Acta Trop..

[7] Ceballos L (2009). Flubendazole in cystic echinococcosis therapy: Pharmaco-parasitological evaluation in mice. Parasitol. Int..

[8] Mackenzie CD, Geary TG (2011). Flubendazole: a candidate macrofilaricide for lymphatic filariasis and onchocerciasis field programs. Expert Rev. Anti-infe..

[9] Canova K, Rozkydalova L, Rudolf E (2017). Anthelmintic flubendazole and its potential use in anticancer therapy. Acta Med..

[10] Králová V, Hanušová V, Rudolf E, Čáňová K, Skálová L (2016). Flubendazole induces mitotic catastrophe and senescence in colon cancer cells in vitro. J. Pharm. Pharmacol..

[11] Hou ZJ (2015). Flubendazole, FDA-approved anthelmintic, targets breast cancer stem-like cells. Oncotarget.

[12] Kralova V (2013). Antiproliferative effect of benzimidazole anthelmintics albendazole, ricobendazole, and flubendazole in intestinal cancer cell lines. Anti-Cancer Drug.

[13] Spagnuolo PA (2010). The antihelmintic flubendazole inhibits microtubule function through a mechanism distinct from Vinca alkaloids and displays preclinical activity in leukemia and myeloma. Blood.

[14] Michaelis M (2015). Identification of flubendazole as potential anti-neuroblastoma compound in a large cell line screen. Sci. Rep..

[15] Fok Eacute, Kassai T (1998). Toxocara canis infection in the paratenic host: a study on the chemosusceptibility of the somatic larvae in mice. Vet. Parasitol..

[16] Chae SW (2011). Overexpressions of cyclin B1, cdc2, p16 and p53 in human breast cancer: the clinicopathologic correlations and prognostic implications. Yonsei. Med. J..

[17] Squires S (2012). Comparative efficacy of flubendazole and a commercially available herbal wormer against natural infections of Ascaridia galli, Heterakis gallinarum and intestinal Capillaria spp. in chickens. Vet. Parasitol..

[18] Tarbiat B (2016). The efficacy of flubendazole against different developmental stages of the poultry roundworm Ascaridia galli in laying hens. Vet. Parasitol..

[19] Ismail KA, Ibrahim AN, Ahmed MAF, Hetta MH (2016). Comparison between the effect of Lawsonia inermis and flubendazole on Strongyloides species using scanning electron microscopy. J. Parasit. Dis..

[20] Purdy D, Aebischer NJ, Davis C (2012). Comparison of single and split-dose flubendazole treatment for the nematode parasite Trichostrongylus tenuis in experimentally infected grey partridges Perdix perdix. Parasitology.

[21] VokŘÁL I (2012). The metabolism of flubendazole and the activities of selected biotransformation enzymes in Haemonchus contortus strains susceptible and resistant to anthelmintics. Parasitology.

[22] Cvilink V (2008). Biotransformation of flubendazole and selected model xenobiotics in Haemonchus contortus. Vet. Parasitol..

[23] BÁRtÍKovÁ H (2010). Flubendazole metabolism and biotransformation enzymes activities in healthy sheep and sheep with haemonchosis. J. Vet. Pharmacol. Ther..

[24] Vuitton DA (1984). Humoral and cellular immunity in patients with hepatic alveolar echinococcosis. A 2 year follow-up with and without flubendazole treatment. Parasite Immunol..

[25] Gomez DE (2012). Telomere structure and telomerase in health and disease. Int. J. Oncol..

[26] Xu C (2013). miRNA-100 inhibits human bladder urothelial carcinogenesis by directly targeting mTOR. Mol. Cancer Ther..

[27] Van Dolah FM, Ramsdell JS (1996). Maitotoxin, a calcium channel activator, inhibits cell cycle progression through the G1/S and G2/M transitions and prevents CDC2 kinase activation in GH4C1 cells. J. Cell. Physiol..

[28] Cicenas J, Valius M (2011). The CDK inhibitors in cancer research and therapy. J. Cancer Res. Clin. Oncol..

[29] White E (1996). Life, death, and the pursuit of apoptosis. Genes Dev..

[30] Fiandalo MV, Kyprianou N (2012). Caspase control: Protagonists of cancer cell apoptosis. Exp. Oncol..

[31] Hensley P, Mishra M, Kyprianou N (2013). Targeting caspases in cancer therapeutics. Biol. Chem..

[32] Jia G (2015). Tubeimoside-1 induces glioma apoptosis through regulation of Bax/Bcl-2 and the ROS/Cytochrome C/Caspase-3 pathway. Onco Targets Ther..

[33] Kaleem S (2016). Eupalitin induces apoptosis in prostate carcinoma cells through ROS generation and increase of caspase-3 activity. Cell. Biol. Int..

[34] Chang GC (2004). Extracellular signal-regulated kinase activation and Bcl-2 downregulation mediate apoptosis after gemcitabine treatment partly via a p53-independent pathway. Eur. J. Pharmacol..

[35] Zhou X (2017). HnRNP-L promotes prostate cancer progression by enhancing cell cycling and inhibiting apoptosis. Oncotarget.

